# Protecting laboratory air quality and comparing clinical assisted reproductive technology cycle outcomes before, during and after wildfire events: a retrospective cohort study

**DOI:** 10.3389/frph.2026.1804327

**Published:** 2026-04-02

**Authors:** Natasha D. Ratnayaka-Gamage, Yujie Cao, Anna Tran, Kate Rainczuk, Jessica M. Stringer, Karla J. Hutt, Kelli Sorby, Amy L. Winship

**Affiliations:** 1 Department of Anatomy and Developmental Biology, Development and Stem Cells Program, Biomedicine Discovery Institute, Monash University, Clayton, VIC, Australia; 2 Number One Fertility Clinic, East Melbourne, VIC, Australia

**Keywords:** bushfire, climate change, environment, fertility, ICSI, IVF, smoke, wildfire

## Abstract

**Introduction:**

Climate change is increasing the frequency and severity of wildfires globally, leading to elevated air pollution and potential human health risks. This study examines whether large-scale wildfires impact indoor air quality within fertility clinics and affect assisted reproductive technology (ART) outcomes.

**Methods:**

A retrospective cohort study of 1,647 ART cycles (IVF and/or ICSI) at Number One Fertility clinics in Victoria, Australia, explored cycles occurring before, during, and after the 2019–2020 ‘Black Summer’ wildfires. ART outcomes were compared across quartiles in 2019–2020 and stratified by maternal age (<38 or ≥38 years). Concentrations of particulate matter (PM_2.5_, PM_10_, and total PM) were measured indoors and outdoors at three clinical sites during and following wildfire exposure.

**Results:**

Results indicated that PM concentrations were consistently lower indoors than outdoors at all clinic locations. Embryology laboratories maintained negligible particulate levels despite elevated outdoor pollution during the wildfires. No significant differences in ART outcomes were observed across cycles performed before, during, or after the wildfire period. Key outcomes such as oocyte yield, fertilisation rates, embryo utilization, clinical pregnancy, and live birth rates remained unaffected.

**Discussion:**

This study highlights the effectiveness of air filtration systems in maintaining air quality within embryology laboratories during wildfire events and emphasizes the importance of such controls in fertility clinics. Future research is needed to investigate the long-term reproductive effects of environmental exposures on fertility, ART cycles and pregnancy outcomes.

## Introduction

The frequency and severity of wildfires, also known as bushfires, is increasing globally due to climate change (1–3). Since 1910, Australia's mean temperature has increased by 1.4 degrees alongside an increase in extreme heat events and a decline in rainfall (4, 5). Australian forests have experienced an increase of over 350% in burned area between 1988 and 2018, which increased further to 800% in 2019 (6). Specifically, the 2019–2020 wildfire season saw the Southern and Eastern parts of Australia experience largescale and intense wildfires, with up to 19 million hectares, or nearly 75,000 square miles of Australian forest and woodlands being burnt (7, 8). Likewise, one study reported that in the U.S, the number of large fires had doubled between 1984 and 2015 due to anthropogenic climate change (9). Given the likely increase in fire weather in the future, it is important to consider how components of wildfire smoke contribute to air pollution, as well as human health.

Wildfire smoke is heavily comprised of particulate matter (PM), volatile organic compounds (VOCs), and polycyclic aromatic hydrocarbons (PAHs) (10). The reproductive systems in both women and men are especially sensitive to genotoxic, oxidative, or inflammatory stress from air pollutants, including fine PM, defined as particles smaller than 2.5 µm (PM_2.5_) (11, 12). Emerging evidence suggests associations between industrial sources of air pollution and impaired fertility and reproductive outcomes. Across human populations, poor air quality and raised levels of PM_2.5_ have been shown to significantly correlate with lower natural fertility rates (13–17), and are also associated with poor outcomes in couples undergoing assisted reproductive technologies (ART) (18–21).

In males, semen parameters are sensitive to exposure to poor air quality (22–24). A systematic meta-analysis found that high levels of air pollution were significantly associated with reduced semen volume, sperm concentration and sperm motility, as well as increased rates of sperm DNA fragmentation in males (25). In females, preclinical studies in rodent models show ovarian follicular atresia following exposure to fine PM_2.5_ compared to ambient levels (26). Currently though, limited information regarding the specific impacts of wildfire exposure on fertility and conception is reported.

Air quality under steady-state conditions in U.S. embryology laboratories has been shown to significantly alter pregnancy outcomes for patients undergoing ART (27). One study reported that ART laboratories with robust air filtration systems resulted in improved air quality metrics and increased fertilisation and live birth rates compared to laboratories without these systems (27). Indeed, exposure to increased air pollution results in impaired embryo development and pregnancy rates, and is ultimately associated with adverse outcomes including miscarriage, pre-term birth and low birth rate (28, 29). Therefore, controlling the air quality in an embryology laboratory is essential to maintaining optimal conditions for embryo culture. The most widely used methods to achieve this involve air pressurisation and high-efficiency particulate air (HEPA) filtration systems. Positive air pressure works by creating differential air pressure in adjacent rooms therefore minimising retention of PM by forcing air out into adjacent spaces (30), whilst HEPA filtration systems remove these particles from the air supply either by diffusion or interception methods (30). Monitoring of air pollutants is not routinely performed in most IVF laboratories but may be a beneficial addition to daily quality assurance. Since PM has been shown to have detrimental effects on the development of embryos in culture and subsequent implantation potential and birth outcomes, it is ideal to monitor air quality levels in the laboratory. However, reports of embryology laboratory air quality measurements are limited in the face of major climactic events that trigger a spike in air pollution, such as wildfires.

Two recent studies examining wildfire occurrence and IVF outcomes have been reported in the literature. A study conducted prior to and during fires in Portland, Oregon, U.S found that the total number of blastocysts obtained during IVF cycles was significantly reduced following exposure to poor laboratory air quality during the 10-day fires, compared to the unexposed cohort (29). Notably, only five days out of the specified ten-day acute exposure period had poor air quality that was classified as hazardous (29). Furthermore, the ten-day acute exposure window resulted in only *n* = 15–16 cycles that corresponded to potential smoke exposure and the limited sample size in this study makes the findings difficult to interpret at the population level. Expanding on this study, Cao et al. recently examined longer-term reproductive outcomes in a retrospective cohort, assessing ART cycles relative to residential proximity to major wildfire events (*n* = 2,001 cycles; six months before and after fires) (31). No significant differences in ART outcomes were observed based on residential proximity; however, correlation analyses suggested a trend toward decreased embryo utilisation rates over time in the residential group directly proximal to wildfires, potentially indicating cumulative reproductive impacts post-fires (31). That study did not measure PM or individual smoke exposure, underscoring the need for future research with direct exposure assessments.

This retrospective cohort study investigated the association between poor air quality from Australian wildfires (November 2019–February 2020) and ART outcomes before, during, and after the fire events. ART outcomes were analysed in patients attending Number One Fertility Clinics in Victoria, Australia (2019–2020), stratified by cycle date and age. Additionally, PM levels were monitored both outdoors and within the embryology laboratory to assess the effectiveness of the laboratory's air-handling systems during the wildfires.

## Materials and methods

### Ethics statement

This study was approved by the Monash University Human Research Ethics Committee (MUHREC #29705). The study was performed in accordance with the ethical standards outlined in the 1964 Declaration of Helsinki and its later amendments.

### Australian wildfire events and proximity to No. 1 fertility clinical sites

Fire events occurred within the State of Victoria during the 2019–2020 Australian summer—which occurred from November through to February. The duration of the fires in the state of Victoria spanned 90 days from 21st November 2019—27th February 2020. The dates of Victorian wildfire events were obtained from the Victorian Government Forest Fire Management website. Three Number One Fertility sites were used for PM measurements and ART outcome assessment—Jolimont, Freemasons (Epworth) and Geelong. Of these, the Jolimont and Freemasons sites are classified as inner city, whilst the Geelong site is classified as a regional area. The 2019–2020 wildfires in Victoria, Australia mainly affected regional and rural areas. However, smoke from these fires travelled thousands of kilometres leading to hazardous air quality within metropolitan Melbourne, even crossing the south Pacific Ocean (32, 33). All three clinics were located ∼100–500 kilometres (or 60–300 miles) away from the occurrence of wildfires.

### Outdoor and indoor laboratory air quality measurements

Three categories of PM were measured daily over a period of two weeks in February 2020, during the height of the Australian wildfire crisis: PM_2.5_, PM_10_ and total PM. Measurements were made using a handheld particle counter (3016-IAQ, Lighthouse Worldwide Solutions, Oregon, USA). PM concentrations were recorded again in the same manner a year later in 2021 during a non-wildfire period to compare the difference between air quality of standard and extreme conditions, at the same time of year. All measurements were taken at the same time daily, during early mornings, prior to high volumes of road traffic (outdoor) or laboratory procedures commencing (indoor).

### Air quality testing sites

PM concentrations were recorded in multiple locations, across multiple Number One Fertility clinic sites. There were three sites across Victoria: Geelong (regional), Freemasons (inner city) and Jolimont (inner city). At each site, measurements were taken at four locations: (1) outdoors, directly outside the building; (2) internally in a public access area, with at least one direct access point to outside i.e., reception or hospital areas (Jolimont and Geelong); (3) inside the day surgery/theatre unit (Freemasons and Geelong) or the andrology laboratory (Jolimont), where these spaces had restricted public access and at least two barriers between the location and the outdoor environment; (4) inside the embryology laboratory.

### Laboratory air handling systems

The embryology laboratories were fitted with a HEPA-filtered positive pressure system, maintaining a pressure differential of 20 pascals in the regional (Geelong) laboratory and 10 pascals in the inner-city laboratories (Freemasons, Jolimont). Laboratories were also fitted with free-standing air filtration units providing activated carbon filtration and photo-catalytic purification.

### Efficacy of portable air purification systems

Two types of portable air purification units, including the Coda Air 800–008 (Cooper Surgical, NSW, Australia) and the ZANDAIR 100 c (Tek-Event Pty Ltd, NSW, Australia) were independently tested for efficacy in a large room (31 m^2^) with entries open to surrounding areas. Initial readings were taken prior to the placement of each unit into the rooms and then again after a period of 24 h (Supplementary Table S1).

### Study population and inclusion/exclusion criteria

This study analysed ART outcomes in women who underwent oocyte retrieval between January 2019—December 2020 at Number One Fertility clinics in Victoria, Australia. Inclusion criteria included clinical infertility treatment, regardless of aetiology. Inclusion criteria also included patients who received sperm donation due to diagnosed male factor infertility, all methods of sperm extraction (ejaculate, testicular, epidydimal and unknown), and all sperm quality factors (concentration of sperm varied from 0.0–400.0 million per millilitre from the ejaculated sample). Patients were excluded from the study if they did not respond to stimulation, had a cancelled oocyte retrieval, froze oocytes in lieu of insemination, donated their oocytes or embryos, received donor oocytes or embryos, or used vitrified oocytes or embryos from previous cycles. Patient demographics, including body mass index (BMI) and infertility diagnosis, are listed in Table 1.

**Table 1:** Patient cohort demographics, including maternal body mass index (BMI), and infertility diagnosis (male factor, endometriosis, tubal factor, other female factor, or unexplained) for patients undergoing oocyte retrieval for IVF and ICSI cycles in 2019/2020.

Parameter	Jan–Mar 2019 (Q1)	Oct–Dec 2019 (Q4)	Jan–Mar 2020 (Q1)	Apr–Jun 2020 (Q2)	Jul–Sep 2020 (Q3)	Oct–Dec 2020 (Q4)
Number of patients (*n*)	242	144	129	333	468	331
Number of women who underwent repeated cycles within the study	19 (7.9%)	3 (2.08%)	6 (4.7%)	29 (8.7%)	60 (12.8%)	26 (7.9%)
Maternal BMI, mean ± SD	23.8 ± 4.3	23.8 ± 4.2	24.4 ± 5.1	24.4 ± 4.7	24.4 ± 5.1	24.2 ± 5.0
Previous 20W pregnancy	39 (16.1%)	33 (22.9%)	25 (19.4%)	70 (21%)	105 (22.4%)	70 (21.1%)
Infertility diagnosis						
Male factor	48 (19.8%)	25 (17.4%)	10 (7.8%)	56 (16.8%)	85 (18.2%)	56 (16.9%)
Endometriosis	49 (20.2%)	26 (18%)	24 (18.6%)	55 (16.5%)	81 (17.3%)	59 (17.8%)
Tubal factor	21 (8.7%)	11 (7.6%)	5 (3.9%)	27 (8.1%)	31 (6.6%)	19 (5.7%)
Other female	85 (35.1%)	50 (34.7%)	50 (38.8%)	132 (39.6%)	204 (43.6%)	147 (44.4%)
Factors Unexplained	76 (31.4%)	54 (37.5%)	49 (38%)	96 (28.8%)	119 (25.4%)	82 (24.8%)

Values are shown as median (range) or % frequency; BMI, body mass index.

### Study cohort and assessment

Female age was reported based on age at oocyte retrieval date. Outcome measures included oocyte number, fertilised oocyte number, fertilisation rate, number of embryos utilised (fertilised embryos that were transferred or vitrified), utilisation rate (number of utilised embryos over number of embryos). Both insemination methods [conventional *in vitro* fertilization (IVF) and intracytoplasmic sperm injection (ICSI)] were included in this study. Clinical pregnancy was defined based upon the Australian and New Zealand Assisted Reproduction Database (ANZARD) criteria which is evidence of a gestational sac, and this was recorded for each transfer cycle. Clinical pregnancy and live birth rates presented per embryo transfer.

### Ovarian stimulation and oocyte retrieval

Ovarian stimulation was performed using gonadotropin-releasing hormone (GnRH) agonist or GnRH antagonist protocols as determined by the clinician based on individual patient history. Human chorionic gonadotropin (hCG) or GnRH agonist (Triptorelin) was used to trigger oocyte maturation, and oocytes were retrieved 36–38 h later. Metaphase II oocytes were inseminated at approximately 39 h post-trigger using intracytoplasmic sperm injection (ICSI) or IVF.

### Sperm preparation

Semen samples were prepared using either Swim-Up or Density Gradient Centrifugation (Sperm Grad, Vitrolife, Gothenburg, Sweden) techniques dependent upon initial sample parameters. Samples for IVF insemination were incubated at 37 °C for 30 min prior to insemination.

### Insemination methodology

IVF insemination was performed by adding 100,000 motile sperm to a 600 µL well of G-IVF PLUS medium (Vitrolife, Gothenburg, Sweden) containing 1–5 oocytes. ICSI was performed in G-1 PLUS medium (Vitrolife, Gothenburg, Sweden) under polarised light, allowing visualisation and positioning (at 11–12 o'clock) of the metaphase spindle at the time of injection.

### Embryo culture, transfer, and freezing

Fertilisation was assessed 16–18 h after insemination. Normal fertilisation was classified by the presence of two pronuclei. All embryos were culture in Embryoscope +timelapse incubators (Vitrolife, Gothenburg, Sweden) at 37 °C with 6% CO_2%_ and 5% O_2_ in Nitrogen. Culture was performed *in vitro*life G-series sequential media containing 5 mg/mL human serum albumin (Vitrolife, Gothenburg, Sweden) under either Ovoil (Vitrolife, Gothenburg, Sweden) or Lifeguard Oil (Cooper Surgical, Trumbull, USA). Single embryo transfer was performed on either day 3 or day 5 (according to specific patient treatment plan), and the remaining embryos were vitrified on days 5–6 using Rapid-i devices and OmniVit or BlastVit (Vitrolife, Gothenburg, Sweden) as per the manufacturer's instructions. Transfers were routinely performed at day 5, with only a small number performed on day 3 due to individual patient preference. Clinical pregnancy data was based upon scans at 6–8 weeks gestation. Live birth data was recorded following delivery, based on direct reporting from the patients, in accordance with reporting requirements.

### Statistical analysis

Normality of the data was assessed using the Shapiro–Wilk test. Air quality data was analysed by Shapiro–Wilk test followed by Dunn's multiple comparisons test (non-parametric data) or Ordinary One-Way ANOVA followed by Holm-Šídák's multiple comparisons test (parametric data). The Wilcoxon rank-sum test was used to analyse non-parametric continuous data (age, number of oocytes collected and fertilised, number of embryos utilised, as well as fertilisation and embryo formation number and rates). Continuous data are presented as mean (SD). Categorical data (clinical pregnancy and live birth outcomes) were analysed using Fisher's exact test. Statistical significance was set at *p* < 0.05.

## Results

**Figure 1:**
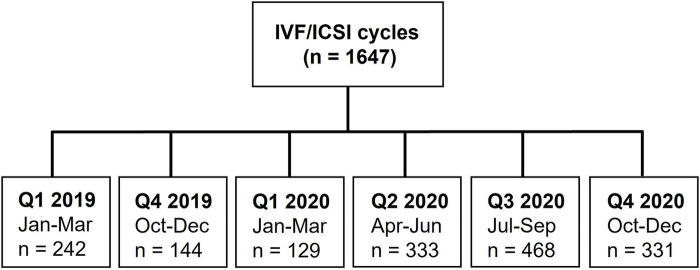
Retrospective study design and patient cohorts included in the study.

This study collected data on ART cycles from 02/01/2019 to 12/12/2020 and overall, 1,647 cycles were included in the analysis (Figure 1). Patient demographics are summarised in Table 1, including number of patients, maternal BMI, previous 20-week pregnancy, as well as infertility diagnosis (male factor, endometriosis, tubal diseases, other female factors, and unexplained).

### Decreasing particulate matter from outdoor to internal laboratory environments

A consistent decrease in PM_2.5_, PM_10_, and total PM was observed from outdoor areas to internal laboratory environments across all three study locations (Table 2). At the Jolimont site (Table 2a), mean outdoor PM_2.5_ levels were 9.91 μg/m^3^ and declined markedly to 0.03 μg/m^3^ in the embryology laboratory (*p* < 0.0001). Outdoor PM_10_ concentrations averaged 79.61 μg/m^3^, decreasing significantly to 8.49 μg/m^3^ in the andrology laboratory (*p* = 0.0253) and 0.91 μg/m^3^ in the embryology laboratory (*p* < 0.0001). Total PM averaged 87.65 µg/m^3^ outdoors and was significantly reduced at reception (16.91 μg/m^3^; *p* < 0.0001), in the andrology laboratory (11.89 μg/m^3^; *p* < 0.0001), and in the embryology laboratory (2.29 μg/m^3^; *p* < 0.0001).

**Table 2:** Air particulate levels measured at various number One fertility sites including jolimont in **a)** 2020 and in **d)** 2021, **b)** Freemasons and **c)** Geelong, in Melbourne Victoria, Australia. Data was analysed by Shapiro–Wilk test followed by Dunn's multiple comparisons test (non-parametric data) or Ordinary One-Way ANOVA followed by Holm-Šídák's multiple comparisons test (parametric data). Statistical significance is set at *p* < 0.05. *p*-value refers to outdoor vs. each indoor location.

a. Jolimont site (2020)					
Air particulate	Average outdoor levels (μg/m^3^)	Average reception levels (μg/m^3^)	Average andrology lab levels (μg/m^3^)	Average embryology lab levels (μg/m^3^)	*p*-value
PM_2.5_	9.92	4.02	2.63	0.03****	****p < 0.0001
PM_10_	79.61	11.72	8.49*	0.91****	*p = 0.0253 ****p < 0.0001
Total PM	87.65	16.91****	11.89****	2.29****	****p < 0.0001

b. Freemasons site (2020)				
Air particulate	Average outdoor levels (μg/m^3^)	Average theatre levels (μg/m^3^)	Average embryology laboratory levels (μg/m^3^)	*p*-value
PM_2.5_	10.05	0.44	0.15***	*p* = 0.0005
PM_10_	69.54	5.30	2.62***	*p* = 0.0008
Total PM	76.39	9.30	5.86***	*p* = 0.0009

c. Geelong (2020)					
Air particulate	Average outdoor levels (μg/m^3^)	Average hospital levels (μg/m^3^)	Average theatre levels (μg/m^3^)	Average embryology laboratory levels (μg/m^3^)	*p*-value
PM_2.5_	10.89	4.56	0.31**	0.01****	**p = 0.0025 ****p < 0.0001
PM_10_	68.28	35.77	2.91**	0.04****	**p = 0.0035 ****p < 0.0001
Total PM	97.57	90.53	6.06*	0.04****	*p = 0.0204 ****p < 0.0001

d. Jolimont (2021)					
Air particulate	Average outdoor levels (μg/m^3^)	Average reception levels (μg/m^3^)	Average andrology lab levels (μg/m^3^)	Average embryology lab levels (μg/m^3^)	*p*-value
PM_2.5_	5.38	2.92	2.79	0.02*	*p* = 0.0298
PM_10_	33.56	9.21	6.09	0.79**	*p* = 0.0025
Total PM	37.56	13.43***	7.14****	1.99****	***p = 0.0002 ****p < 0.0001

At the Freemasons clinic (Table 2b), outdoor PM_2.5_ averaged 10.05 μg/m^3^ and decreased significantly to 0.15 μg/m^3^ within the embryology laboratory (*p* = 0.0005). Outdoor PM_10_ levels averaged 69.54 μg/m^3^, dropping to 2.62 μg/m^3^ indoors (*p* = 0.0008). Total PM also declined from 76.39 μg/m^3^ outdoors to 5.8 μg/m^3^ in the embryology laboratory (*p* = 0.0009).

At the Geelong site (Table 2c), outdoor PM_2.5_ averaged 10.89 μg/m^3^. Concentrations were significantly lower in both the theatre (0.31 μg/m^3^; *p* = 0.0025) and the embryology laboratory (0.01 μg/m^3^; *p* < 0.0001). Outdoor PM_10_ levels averaged 68.28 μg/m^3^ and declined to 2.91 μg/m^3^ in the theatre (*p* = 0.0025) and 0.04 μg/m^3^ in the embryology laboratory (*p* < 0.0001). Total PM averaged 97.57 μg/m^3^ outdoors, decreasing to 6.06 μg/m^3^ in the theatre (*p* = 0.0204) and further to 0.04 μg/m^3^ in the embryology laboratory (*p* < 0.0001). Reductions at this site likely reflect restricted access and enhanced air-filtration systems.

Air quality data collected at Jolimont during a non-wildfire period (2021) (Table 2d) showed lower levels compared to the wildfire period, and a similar pattern of decline in PM levels from outdoor to indoor environments. Outdoor PM_2.5_ averaged 5.38 μg/m^3^, decreasing to 0.02 μg/m^3^ in the embryology laboratory (*p* = 0.0298). Outdoor PM_10_ averaged 33.56 μg/m^3^ and declined to 0.79 μg/m^3^ indoors (*p* = 0.0025). Total PM decreased from 37.56 μg/m^3^ outdoors to 13.43 μg/m^3^ at reception (*p* = 0.0002), 7.14 μg/m^3^ in the andrology laboratory, and 1.99 μg/m^3^ in the embryology laboratory (*p* < 0.0001).

### Clinical outcomes for IVF and ICSI cycles before, during, and after wildfire events

To evaluate whether wildfire exposure influenced ART outcomes, ART parameters were compared between patients who presented during Q1 2019 (non-wildfire period) and Q1 2020 (peak wildfire period) (Table 3). No significant differences were detected between these groups in mean patient age, oocyte yield, number of oocytes fertilised, fertilisation rate, embryos utilised, or embryo utilisation rate.

**Table 3:** IVF cycle (conventional + ICSI) outcomes for women undergoing oocyte retrieval. Data was stratified based on 2019 & 2020 divided into quartiles (Q1), and further stratified based on age (<38 years or ≥38 years). Data are presented as mean ± SD, analysed via Wilcoxon rank-sum test with statistical significance set at *p* < 0.05.

Parameter		Jan–Mar 2019 (Q1)	Jan–Mar 2020 (Q1)
Mean age of female, mean ± SD	All ages	37.4 ± 4.1	37.7 ± 4.0
	<38	34.0 ± 2.5	34 ± 2.5
	≥38	40.7 ± 2.2	40.6 ± 2.2
Mean no. of oocytes collected, mean ± SD	All ages	9.0 ± 6.0	10 ± 7.3
	<38	11 ± 6.5	12 ± 7.8
	≥38	8.0 ± 5.0	9 ± 6.6
Mean no. of oocytes fertilised, mean ± SD	All ages	4.0 ± 3.4	4 ± 3.5
	<38	5.0 ± 3.8	5 ± 4.0
	≥38	3.0 ± 2.8	3 ± 2.9
Fertilisation rate (%), mean ± SD	All ages	56.0 ± 28.9	50.5, ±27.1
	<38	55.0 ± 28.2	55.3 ± 26.6
	≥38	56.9 ± 29.6	46.6 ± 27.1
Mean no. of embryos utilized, mean ± SD	All ages	2.0 ± 2.1	2 ± 2.1
	<38	2.0 ± 2.5	3 ± 2.3
	≥38	1.0 ± 1.6	2 ± 1.7
Embryo utilisation rate (%), mean ± SD	All ages	46.4 ± 33.3	53.3 ± 33.9
	<38	53.0 ± 30.8	57.4 ± 31.9
	≥38	39.9 ± 34.6	50 ± 35.4

To determine whether ART outcomes were impacted over time post-fires, data were stratified according to the quarter in which patients presented to the clinic throughout 2020 (Q1–Q4), with Q1 representing the peak wildfire period and Q2–4 representing post-fire periods. No significant differences were observed across these quartiles in mean patient age, number of oocytes retrieved, number of oocytes fertilised, fertilisation rate, number of embryos utilised, or embryo utilisation rate (Table 4).

**Table 4:** IVF cycle (conventional + ICSI) outcomes for women undergoing oocyte retrieval. Data was stratified based on 2020 divided into quartiles (Q1–4), and further stratified based on female age (<38 years or ≥38 years). Data are presented as mean ± SD, analysed via Wilcoxon rank-sum test with statistical significance set at *p* < 0.05.

Parameter		Jan–Mar 2020 (Q1)	Apr–Jun 2020 (Q2)	Jul–Sep 2020 (Q3)	Oct–Dec 2020 (Q4)
Mean age of female, ± SD	All ages	37.7 ± 4.0	36.9 ± 4.5	37.12 ± 4.0	37.23 ± 4.0
	<38	34 ± 2.5	33.4 ± 3.1	33.9 ± 2.5	34 ± 2.6
	≥38	40.6 ± 2.2	40.6 ± 2.1	40.5 ± 1.9	40.5 ± 1.9
Mean no. of oocytes collected, mean ± SD	All ages	10 ± 7.3	11 ± 7.1	11 ± 7.5	10 ± 7.3
	<38	12 ± 7.8	13 ± 7.6	13 ± 8.1	12 ± 7.4
	≥38	9 ± 6.6	9 ± 5.9	9 ± 6.2	9 ± 6.9
Mean no. of oocytes fertilised, mean ± SD	All ages	4 ± 3.5	4 ± 4.0	5 ± 4.7	5 ± 4.3
	<38	5 ± 4.0	5 ± 4.3	6 ± 4.9	6 ± 4.6
	≥38	3 ± 2.9	3 ± 3.4	4 ± 4.3	3 ± 3.7
Fertilisation rate (%), mean ± SD	All ages	50.5, ±27.1	48.5 ± 25.9	48.9 ± 28.1	47.35 ± 26.9
	<38	55.3 ± 26.6	50.6 ± 24.4	53.7 ± 24.8	51.8 ± 24.4
	≥38	46.6 ± 27.1	46.2 ± 27.4	43.9 ± 30.5	42.8 ± 28.7
Mean no. of embryos utilized, mean ± SD	All ages	2 ± 2.1	2 ± 2.5	3 ± 2.9	2 ± 2.8
	<38	3 ± 2.3	3 ± 2.7	4 ± 3.0	3 ± 3.4
	≥38	2 ± 1.7	2 ± 2.0	2 ± 2.7	1 ± 1.7
Embryo utilisation rate (%), mean ± SD	All ages	53.3 ± 33.9	53.8 ± 33.1	54, ±31.0	53 ± 32.0
	<38	57.4 ± 31.9	58.6 ± 29.6	57.7 ± 29.4	56.9 ± 29.5
	≥38	50 ± 35.4	48.4 ± 36.0	49.5 ± 32.3	48.5 ± 34.3

To account for potential seasonal variation in clinic attendance and ART outcomes, particularly towards the end of the year, ART outcomes were further compared between Q4 2019 and Q4 2020 (Table 5). Similar to earlier comparisons, mean patient age, oocyte number, fertilisation metrics, and embryo utilisation measures did not differ significantly between the two periods.

**Table 5:** IVF cycle (conventional + ICSI) outcomes for women undergoing oocyte retrieval. Data was stratified based on 2019 & 2020 divided into quartiles (Q1–4), and further stratified based on age (<38 years or ≥38 years). Data are presented as mean ± SD, analysed via Wilcoxon rank-sum test with statistical significance set at *p* < 0.05.

Parameter		Oct–Dec 2019 (Q4)	Oct–Dec 2020 (Q4)
Mean age of female, mean ± SD	All ages	37.7 ± 4.2	37.23 ± 4.0
	<38	34.1 ± 2.4	34 ± 2.6
	≥38	41 ± 2.3	40.5 ± 1.9
Mean no. of oocytes collected, mean ± SD	All ages	9 ± 5.9	10 ± 7.3
	<38	10 ± 6.0	12 ± 7.4
	≥38	8 ± 5.6	9 ± 6.9
Mean no. of oocytes fertilised, mean ± SD	All ages	4 ± 3.9	5 ± 4.3
	<38	5 ± 4.3	6 ± 4.6
	≥38	3 ± 3.3	3 ± 3.7
Fertilisation rate (%), mean ± SD	All ages	51 ± 28.1	48.9 ± 28.1
	<38	58.9 ± 26.2	51.8 ± 24.4
	≥38	44.1 ± 28.1	42.8 ± 28.7
Mean no. of embryos utilized, mean ± SD	All ages	2 ± 2.4	2 ± 2.8
	<38	3 ± 2.6	3 ± 3.4
	≥38	1 ± 2.1	1 ± 1.7
Embryo utilisation rate (%), mean ± SD	All ages	52.0 ± 34.3	53 ± 32.0
	<38	54.9 ± 30.2	56.9 ± 29.5
	≥38	49.2 ± 38.0	48.5 ± 34.3

### Effects of female age

Given that maternal age may affect ART outcomes (34, 35), this analysis was further subdivided into two groups classified as either young maternal age (<38 years) or advanced maternal age (≥38 years) at the time of oocyte collection (Tables 3–5). The mean age of the patients, as well as the mean number of oocytes collected, fertilised, fertilisation rate, mean number of embryos utilised, and embryo utilisation rate did not significantly differ between quartiles, and this did not change with the age stratification.

### Pregnancy outcomes

No significant differences were detected in clinical pregnancy outcomes or live births, based on cleavage and blastocyst stage transfers, between patients presenting during the Q1–Q4 2020, and Q1 vs. Q4 2019 (Table 6).

**Table 6:** Pregnancy and live birth outcomes in patients undergoing oocyte retrieval before or after the 2019–2020 wildfire events for all ages. Data was stratified based on 2019 & 2020 divided into quartiles (Q1–4). Data are presented as percentages, analysed via Chi square or Fisher's exact test with statistical significance set at *p* < 0.05.

Parameter	Q1 2019	Q4 2019	Q1 2020	Q2 2020	Q3 2020	Q4 2020
Number of fresh transfers	54	44	32	118	168	133
Mean age of female ± SD	37.4 ± 4.1	37.7 ± 4.2	37.7 ± 4.0	36.9 ± 4.5	37.12 ± 4.0	37.23 ± 4.0
Clinical pregnancy per transfer	18/54 (33.3%)	15/44 (34.1%)	11/32 (34.4%)	44/118 (37.3%)	69/168 (41%)	46/133 (34.6%)
Cleavage stage transfer	0/18 (0%)	1/15 (6.7%)	0/11 (0%)	0/44 (0%)	0/69 (0%)	0/46 (0%)
Blastocyst stage transfer	18/18 (100%)	14/15 (93.3%)	11/11 (100%)	44/44 (100%)	69/69 (100%)	46/46 (100%)
Live birth per transfer	14/54 (25.9%)	13/44 (29.5%)	9/32 (28.1%)	35/118 (29.7%)	52/168 (31%)	33/133 (24.8%)
Cleavage stage transfer	0/14 (0%)	0/13 (0%)	0/9 (0%)	0/35 (0%)	0/52 (0%)	0/33 (0%)
Blastocyst stage transfer	14/14 (100%)	13/13 (100%)	9/9 (100%)	35/35 (100%)	52/52 (100%)	33/33 (100%)

## Discussion

Previous studies have demonstrated that exposure to wildfire smoke during pregnancy is associated with increased risk of adverse pregnancy outcomes (36–40), however, clear evidence regarding the potential impact of wildfire smoke exposure on pre-conception fertility remains limited. Here, we report air quality levels measured across multiple sites including outdoor and indoor sites from numerous Number One Fertility clinics in Victoria, Australia, during and after the 2019/2020 wildfire period. We also examined the potential influence of environmental exposure to wildfire smoke on clinical ART outcomes before, during, and after the wildfires. We observed a significant reduction in PM_2.5_, PM_10_ and total PM levels progressively as facility access became more restricted, reaching their lowest values within the embryology laboratories where ART procedures were performed. Concurrent with these findings, ART outcomes remained unchanged, with the number of oocytes retrieved, number of oocytes fertilised, fertilisation rate, number of embryos utilised, and rate of embryo utilisation remaining similar among patients presenting to the clinics during various quartiles before, throughout, and after the fires. Taken together these findings suggest that whilst wildfire events may substantially reduce overall ambient outdoor air quality, conditions within ART facilities with appropriate air filtration measure in place remain stable. These findings underscore the efficacy of existing air-filtration systems and reinforce the importance of monitoring and maintaining optimal air quality in these settings.

In support of the current findings, a large retrospective cohort study by Boulet et al. (41) involving more than 200,000 women, reported no significant difference in IVF outcomes in relation to elevated outdoor PM_2.5_ levels (41). Similarly, a recent study by Cao et al. (31) assessed reproductive outcomes in a retrospective cohort of ART cycles relative to residential proximity to major wildfire events (31). Although no significant differences in ART outcomes were detected based on proximity, correlation analyses suggested a trend toward declining embryo utilisation rates over time in the proximal residential group, possibly indicating a decrease in gamete quality in the months following wildfire exposure (31). However, that study did not quantify laboratory or ambient PM, nor individual smoke exposure, limiting the interpretation of the outcomes.

In contrast, Kornfield et al. (29) reported a significant reduction in the number of blastocysts obtained among patients undergoing IVF during an acute wildfire event compared with unexposed patients, although fertilisation rates and pregnancy outcomes were unaffected (29). Notably, PM_2.5_ levels differed vastly between these studies; Boulet et al. (41) reported median PM_2.5_ levels ranging from 9.1–9.5 µg/m^3^ on the day of embryo transfer (41), whereas Kornfield et al. (29), documented levels as high as 465 µg/m^3^ during the wildfire season (29), although individual patient exposures were not quantified. These discrepancies emphasise the need for further investigation to decipher whether the differences in ART outcomes under elevated PM_2.5_ exposure are attributed to variation in filtration system capabilities, highlighting the critical importance of establishing and sustaining appropriate air filtration infrastructure in spaces where ART is performed. This also raises important questions regarding how pre-clinical exposure to environmentally relevant levels of wildfire-derived PM affects ART outcomes, regardless of air filtration efficacy in embryology laboratories.

In the current study, we were unable to incorporate analysis based on residential proximity to wildfires, as preliminary data stratification revealed that the patient cohort largely resided in metropolitan inner-city areas. Despite the huge scale of the 2019/2020 Australian “Black Summer” wildfires, these fires occurred in regional areas with lower population, when compared with metropolitan and suburban areas within the state, meaning that the statistical power to compare differences between proximal and non-proximal groups before and after fires was greatly reduced. As the findings from the current study are difficult to interpret in terms of individual patient exposure to PM and the subsequent impacts on ART success, this indicates that further studies are warranted. In particular, *in vitro*, *ex vivo* and *in vivo* studies should be established to model wildfire effects on fertility and to uncover the potential mechanistic impacts.

A confounding variable is the overlap between the current study (2019 and 2020 quartiles) with the COVID-19 pandemic. One major COVID-19 related change may include a patient selection based on a national pause of elective procedures including IVF and ICSI, resulting in selection bias and potential changes in patient demographics during the COVID-19 period, and full reopening of clinics later on. The subsequent changes in clinical practice, including reduced on-site staffing, increased telehealth consultations, altered embryologist schedules, and a potential increase in frozen cycles may have impacted ART outcomes including fertilisation and blastocyst rates, and the timing of embryo transfers. Furthermore, the potential prolonged psychological stress resulting from severe lockdown and social isolation practices, as well as the COVID-19 related clinic changes may have had subsequent impacts. The COVID-19 infection in itself may have had effects on female reproductive capacity, as alluded to by one retrospective cohort study comparing 4,099 cycles from women with a positive COVID-19 diagnosis with 6,041 age-matched COVID-19 negative controls, finding that the miscarriage rate was higher in patients undergoing ART treatment <30 days following COVID-19 infection compared to COVID-19 negative controls (42). Although the peak wildfire exposure period preceded the suspension of ART services in late March 2020, the analysis spanned throughout the entirety of 2020. The data should be interpreted with caution given the potential for the confounding influence of COVID-19 mediated healthcare disruptions throughout 2020.

In males, recent literature has reported an association between wildfire smoke exposure and altered semen analysis parameters. Several studies have reported a decrease in motile sperm count and/or concentration in response to poor air quality from wildfire smoke, which could be an indication of compromised sperm quality (43–46). Indeed, one study reported an association between high levels of air pollution exposure and percentage of sperm with DNA fragmentation (47). Research in preclinical animal models has revealed that chronic exposure to wildfire smoke induces epigenetic modifications in mouse sperm, by disrupting DNA methylation patterns (48). These data highlight sperm as a sensitive biomarker following wildfire associated particulate exposure and a potentially significant contributor to altered ART outcomes.

In females, several studies in preclinical animal reported that the ovarian reserve and live birth rates are lower following PM_2.5_ exposure (49–51). Given the array of potential mechanisms by which wildfire exposure, primarily the subsequent PM_2.5_ exposure, may interfere with fertilisation through affecting male and female reproductive parameters, it is difficult to distinguish between male and female factor specific contributions to the findings of the current study. In females, it is possible that environmental insults, such as atmospheric contaminants during and after wildfires, affect oocyte number and quality, particularly the finite primordial follicle reserve. Such effects may not be immediately detectable in ART cycle analyses, including those presented here. A Danish cohort study spanning 17 years and nearly 380,000 women reported an increased risk of infertility among women aged 35–45 following long-term exposure to traffic-related PM_2.5_ (>12 μg/m³) (52). Similarly, long-term U.S. mortality data identified associations between wildfire exposure and more than 11,000 deaths up to 13 years post-exposure (53). These findings suggest that health impacts may manifest over considerably longer periods than those assessed in this study. Preclinical evidence supports this concern, as PM_2.5_ exposure depletes the ovarian reserve in adult mice (54), and maternal PM_2.5_ exposure reduces ovarian reserve endowment in neonatal female offspring (55). Future work should therefore investigate the longer-term impacts specific wildfire smoke constituents, including but not limited to PM_2.5_, on fertility and the reproductive lifespan in both clinical studies and preclinical models.

## Conclusions

In this study, we examined the association between the 2019/2020 wildfires—resulting in changes in ambient air quality—and clinical ART outcomes. Concentrations of PM_2.5_, PM_10_, and total PM were markedly reduced from outdoor to indoor environments within the clinics, with the greatest reduction recorded within embryology laboratories. Notably, no significant differences in ART outcomes were evident among patients presenting before, during, or after the wildfire events. These findings emphasise the value of rigorous air-quality monitoring and mitigation strategies in ART settings. Nevertheless, further investigation is warranted to clarify the potential impacts of environmental wildfire smoke exposure on reproductive success. Future research should include prospective study designs incorporating accurate exposure assessment, as well as mechanistic investigations using *in vitro* and *in vivo* models to elucidate the biological pathways through which fire-derived pollutants may affect reproductive function. More broadly, our findings highlight the importance of effective air-quality management and public health guidance during wildfire events, particularly for those planning to conceive.

## Data Availability

The original contributions presented in the study are included in the article/Supplementary Material, further inquiries can be directed to the corresponding author.
