# Supplementary material for: Protecting laboratory air quality and comparing clinical assisted reproductive technology cycle outcomes before, during and after wildfire events: a retrospective cohort study
Source: Front Reprod Health. 2026 Apr 2;8:1804327. doi: 10.3389/frph.2026.1804327 (PMC13083208; doi:10.3389/frph.2026.1804327)
Supplement: Supplementary file 1 [file Table1.docx]

**Supplementary table 1:** Efficacy of Portable Air Purification Systems tested using Coda Air 800-008 and ZANDAIR 100c portable purification units within the No.1 Fertility clinics with measures pre and post 24-hours. Particulate levels were compared between pre- and post-24-hour average measurements for small and large spaces.

a. CodaAir air purification system

| **Air particulate** | **Average levels before (small space) (**μ**g/m^3^)** | **Average levels after (small space) (**μ**g/m^3^)** |
| --- | --- | --- |
| PM_2.5_ | 2.60 | 1.09 |
| PM_10_ | 20.74 | 2.87 |
| Total PM | 26.67 | 3.64 |

b. ZANDAIR air purification system

| **Air particulate** | **Average levels before (small space) (**μ**g/m^3^)** | **Average levels after (small space) (**μ**g/m^3^)** |
| --- | --- | --- |
| PM_2.5_ | 4.52 | 1.21 |
| PM_10_ | 9.24 | 3.17 |
| Total PM | 10.89 | 4.14 |
